# Reciprocal Within-Person Dynamics Between Internet Gaming Disorder Symptoms, Physical Activity, and Loneliness Among Chinese Adolescent Gamers: Three-Wave Prospective Cohort Study

**DOI:** 10.2196/87847

**Published:** 2026-04-27

**Authors:** Chang Hu, Xuan Meng, Xi Wang, Jilin Huang

**Affiliations:** 1 Professional Tennis College Wuhan City Polytechnic Wuhan, Hubei China; 2 School of Physical Education Jiangxi Normal University Nanchang, Jiangxi China; 3 Nanxishan Hospital of Guangxi Zhuang Autonomous Region Guilin, Guangxi China; 4 General Education Department Wuhan College Wuhan, Hubei China; 5 Faculty of General Education Hubei College of Chinese Medicine Jingzhou, Hubei China

**Keywords:** Chinese adolescents, sexual differences, internet gaming disorder symptoms, loneliness, reciprocal association, physical activity

## Abstract

**Background:**

Problematic gaming and internet gaming disorder (IGD) symptoms are prevalent in adolescence, yet the longitudinal interplay between physical activity (PA), loneliness, and IGD symptoms, as well as potential sexual differences, remains unclear.

**Objective:**

This study aimed to examine reciprocal within-person associations between PA, loneliness, and IGD symptoms among Chinese adolescent gamers and test sexual differences in these associations.

**Methods:**

We conducted a three-wave prospective cohort study among 1332 Chinese adolescents selected using convenience sampling from five middle schools in central China who had engaged in online gaming during the previous year (n=441, 33.1%, females; mean age 3.64, SD 0.76, years). PA, loneliness, and IGD symptoms were assessed using the Physical Activity Rating Scale-3, the three-item short form of the University of California, Los Angeles Loneliness Scale, and the nine-item *Diagnostic and Statistical Manual of Mental Disorders, Fifth Edition* IGD Checklist, respectively, at three 6-month intervals: wave 1 (baseline, T1), wave 2 (T2), and wave 3 (T3). A random intercept (RI) cross-lagged panel model (CLPM) and multigroup analyses were conducted.

**Results:**

RI-CLPM indicated that at the within-person level, higher PA predicted decreased subsequent loneliness (T1→T2: β=–0.12, 95% CI −0.16 to −0.08; T2→T3: β=–0.13, 95% CI −0.17 to −0.08) and IGD symptoms (T1→T2: β=–0.10, 95% CI −0.16 to −0.04, P=.009; T2→T3: β=–0.10, 95% CI −0.16 to −0.04). Increased loneliness (β=–0.22, 95% CI −0.26 to −0.18) and increased IGD symptoms (β=–0.08, 95% CI −0.12 to −0.04) each predicted later reductions in PA, indicating a mutually reinforcing cycle. Increased loneliness also predicted an increase in subsequent IGD symptoms (T1→T2: β=0.14, 95% CI 0.11-0.17), and increased IGD symptoms, in turn, predicted greater loneliness in the next wave (T1→T2: β=0.18, 95% CI 0.14-0.22). Multigroup models indicated that the protective effect of PA on later loneliness and the prospective effect of loneliness on subsequent IGD symptoms were stronger among girls than boys. In males, PA significantly predicted loneliness (β=–0.08 to –0.09, 95% CI −0.15 to −0.01), and loneliness significantly predicted IGD symptoms (β=0.09, 95% CI 0.03-0.15). In females, the cross-lagged effects from PA to loneliness were significant but stronger (β=–0.17, 95% CI −0.23 to −0.11), and the paths from loneliness to later IGD symptoms were also significant (β=0.16, 95% CI 0.11-0.21).

**Conclusions:**

PA, loneliness, and IGD symptoms are reciprocally linked in adolescent gamers. By using RI-CLPM to distinguish within-person changes from stable between-person differences, this study extends prior research based mainly on cross-sectional designs or traditional CLPMs and provides a clearer understanding of the dynamic interplay among behavioral, emotional, and gaming-related factors. The findings highlight that interventions aiming to prevent IGD symptoms should simultaneously promote PA and reduce loneliness, with particular attention to sex-specific patterns, especially in girls.

## Introduction

### Background

Internet gaming disorder (IGD) is a persistent and maladaptive pattern of online gaming associated with significant functional impairments and is increasingly recognized as a major public health concern [1]. IGD is characterized by impaired control over gaming, prioritization of gaming over other activities despite negative consequences, and persistent gaming behavior despite functional impairment [2]. In population-based research, these manifestations are commonly examined in terms of IGD symptoms, which reflect the severity of problematic gaming behaviors [3-7]. Higher levels of IGD symptoms have been linked to a range of detrimental outcomes, including psychological difficulties, behavioral problems, and social consequences [8-11]. Given the widespread accessibility of online games and the developmental vulnerability of adolescence [12,13], understanding the longitudinal mechanisms underlying IGD symptoms and identifying relevant risk and protective factors during this period is particularly important. However, the dynamic interplay among behavioral and emotional factors related to IGD symptoms remains insufficiently understood.

### Longitudinal Association Between Physical Activity and IGD Symptoms

The displacement hypothesis posits that time is limited, so engagement in one activity reduces the time available for others [14]. In this study, this mainly concerned adolescents’ leisure time outside of structured school activities. In Chinese schools, mobile phone use during class is restricted, and gaming typically occurs after school or during other leisure periods [15]. Therefore, greater engagement in physical activity (PA) may reduce opportunities for extended gaming and, in turn, lower the risk for IGD symptoms [16,17]. In addition, PA may promote healthier lifestyle routines, better emotional regulation, and alternative sources of enjoyment and social interaction, thereby reducing adolescents’ reliance on gaming as a primary leisure activity [18-20]. At the same time, this relationship may be bidirectional. Excessive gaming or more severe IGD symptoms may reduce participation in PA by consuming leisure time, lowering motivation for physical movement, and disrupting daily routines or social patterns [21-23]. Taken together, these mechanisms suggest a potentially bidirectional relationship between PA and IGD symptoms.

Accumulating empirical evidence indicates a negative association between PA and IGD symptoms. Cross-sectional investigations across diverse cultural contexts, such as Australia, Taiwan, Portugal, Germany, and China, have consistently shown that higher PA is associated with decreased IGD symptoms [16,17,24-26]. Systematic reviews and meta-analyses have further indicated that exercise-based interventions can reduce gaming addiction scores and may be among the most effective interventions for adolescent internet addiction [18,27]. Longitudinal findings have provided further, although mixed, evidence. A Swiss cohort study found bidirectional associations between video gaming disorder symptoms and sport or exercise participation over 15 months [22], and another longitudinal study reported that higher PA is associated with decreased IGD symptoms [28]. However, other studies have found that IGD symptoms predict later PA but PA does not predict later IGD symptoms [29] or that gaming time predicts PA trajectories but not vice versa [30]. Notably, most prior longitudinal studies have relied on the traditional cross-lagged panel model (CLPM), which cannot disentangle within-person fluctuations from stable between-person differences [31]. Consequently, the within-person longitudinal association between PA and IGD symptoms remains insufficiently understood.

### Longitudinal Association Between Loneliness and IGD Symptoms

Loneliness is a distressing emotional state arising from the perceived discrepancy between desired and actual social relationships [32]. Rather than reflecting objective social isolation, it represents a subjective sense of lacking meaningful connections with others [33]. According to the model of compensatory internet use [34], negative life situations may motivate individuals to go online to regulate negative emotions, and problematic online behaviors arise from attempts to compensate for adverse offline experiences [34]. Online gaming may temporarily relieve loneliness by providing social interaction, achievement, and escape from a negative affect [35]. However, when such compensatory use becomes habitual, it may develop into maladaptive patterns characteristic of increased IGD symptoms [36]. Over time, reliance on gaming as a coping strategy may reduce opportunities for meaningful offline interactions and further intensify loneliness [33]. Likewise, increased IGD symptoms may increase loneliness by reducing real-life social engagement, substituting online interactions for offline relationships, and fostering social withdrawal [37]. These mechanisms suggest a dynamic, bidirectional relationship between loneliness and IGD symptoms.

Empirical studies generally support this perspective. Cross-sectional studies across university and adolescent samples have consistently shown a positive association between loneliness and IGD symptoms [38-42]. Meta-analytic evidence has further identified loneliness as a risk factor of IGD symptoms and internet use disorder symptoms more broadly [43,44]. Longitudinal research has also provided support. Baseline loneliness significantly predicted later IGD symptoms among Chinese university students [45], loneliness predicted increases in gaming problems at both within-person and between-person levels [46], and a two-wave longitudinal study of Dutch adolescent gamers identified loneliness to be both an antecedent and a consequence of pathological gaming [47]. However, most longitudinal studies have used traditional structural equation modeling (SEM) or CLPM, both of which are limited in distinguishing within-person fluctuations from stable between-person differences [48]. As a result, the dynamic longitudinal association between loneliness and IGD symptoms remains insufficiently understood. Furthermore, much of the existing evidence focuses on general internet addiction rather than IGD symptoms specifically, and research among adolescent gamers remains relatively scarce.

### Longitudinal Association Between PA and Loneliness

The bidirectional association between PA and loneliness can be understood within the framework of basic psychological needs theory (BPNT), a subtheory of self-determination theory [49]. BPNT posits that the fulfillment of three innate psychological needs, namely autonomy, competence, and relatedness, is essential for psychological well-being [50,51]. In this context, PA may help satisfy the need for relatedness by providing opportunities for social interaction, belonging, and shared achievement [52]. Regular participation in exercise or group-based sports may thus alleviate loneliness through enhanced social connectedness and emotional support [53]. Conversely, persistent loneliness may undermine these needs by reducing social motivation and self-regulatory capacity, thereby decreasing engagement in PA over time [54]. This reciprocal process suggests that PA and loneliness may influence each other dynamically across time.

Empirical findings generally support this proposition. Cross-sectional research consistently reveals a negative association between PA and loneliness across diverse populations [55-57]. Systematic reviews support this claim [58,59]. Longitudinal evidence further reports the bidirectional association between PA and loneliness among adults and older adults [60,61]. A recent study using a random intercept cross-lagged panel model (RI-CLPM) further demonstrated small but significant within-person bidirectional effects between PA and loneliness [62]. However, most existing longitudinal studies have focused on adult or older populations, with adolescents receiving far less attention. Additionally, many studies have relied on traditional CLPMs. Moreover, relatively few studies have simultaneously examined PA, loneliness, and IGD symptoms within an integrated longitudinal framework. To address these limitations, this study used an RI-CLPM to clarify the bidirectional relationship between PA and loneliness among adolescents.

### Potential Sexual Differences

Emerging evidence suggests that sex may moderate the associations among PA, loneliness, and IGD symptoms. Epidemiological studies consistently show that male adolescents have a higher prevalence of IGD than females [63,64], and males with more severe IGD symptoms appear to be more sensitive to gaming-related rewards, whereas females tend to exhibit stronger affective dysregulation and mood-related symptoms [64]. Sexual differences are also evident in PA levels, with males generally reporting higher engagement in PA than females [65]. Notably, when PA levels are low, males report slightly higher internet addiction than females, but as PA increases, males show a sharper decline in addiction symptoms, suggesting that the protective role of PA may be particularly pronounced among males [65]. At the same time, loneliness and its correlates may operate differently across sexes. Prior studies have shown that the correlation between loneliness and internet addiction tends to be weaker among males [30]. Furthermore, longitudinal evidence shows that moderate-to-vigorous PA negatively predicts gaming time among males but not females [30]. Moreover, sexual differences have been observed in the link between PA and psychological distress [66], where the negative association between PA and loneliness is more pronounced among females [67]. These findings indicate that sex may moderate the associations among PA, loneliness, and IGD symptoms. However, few longitudinal studies have examined whether such sex-specific patterns remain stable over time in youth populations.

### Aims of This Study

To address these gaps, this study adopted a three-wave longitudinal design and an RI-CLPM to examine the temporal associations between PA, loneliness, and IGD symptoms among Chinese adolescent gamers. By distinguishing stable between-person differences from within-person fluctuations, this approach clarified the dynamic reciprocal relationships among these variables over time. Four hypotheses were proposed:

- Hypothesis (H)1: PA and IGD symptoms are reciprocally associated.
- H2: Loneliness and IGD symptoms are reciprocally associated.
- H3: PA and loneliness are reciprocally associated.
- H4: These within-person reciprocal associations differ between male and female adolescents.

## Methods

### Study Design

This 1-year longitudinal study was conducted from March 2024 to March 2025, with data collected at three 6-month intervals: wave 1 (baseline [T1], March 2024), wave 2 (T2, September 2024), and wave 3 (T3, March 2025).

### Participants and Procedures

#### Inclusion and Exclusion Criteria

The target population comprised adolescents who had engaged in online gaming during the preceding 12 months, thereby ensuring that all participants were active gamers rather than nongaming peers. Eligible participants were students from grades 7 and 8. Grade 9 students were excluded because they typically prepare for the high school entrance examination and may graduate or transfer to different schools, which would make longitudinal follow-up difficult. Additionally, focusing on grades 7 and 8 allowed us to capture adolescents in early adolescence, a critical developmental stage for the emergence of problematic gaming behaviors and social-emotional changes [11,68].

#### Sampling Procedures

The study was conducted in a school-based educational setting, and all participating schools were urban public middle schools located in central China. Participants were recruited using convenience sampling in collaboration with school administrators, and recruitment procedures included announcements in classrooms and direct invitations by trained field researchers.

#### Participant Characteristics

At baseline (T1), 1332 adolescents (n=441, 33.1%, females; mean age 13.64, SD 0.76, years) who met the inclusion criteria completed the survey. By T2, 1240 (93.1%) students had contributed two waves of data. By the final wave (T3), 1138 students remained in the study, yielding a retention rate of 85.4%. Participant attrition across waves was mainly attributable to common circumstances in school-based longitudinal surveys, such as student absence on the day of data collection, transfers to other schools during the follow-up period, or incomplete questionnaires that could not be matched across waves.

#### Sample Size, Power, and Precision

The sample size was determined using the number of eligible students available in the participating schools during the recruitment period. Given the longitudinal design and the use of an RI-CLPM, the available sample size was considered adequate to support estimation of within-person longitudinal associations. In addition, all participants who provided valid data at least once were retained in the main analyses to maximize statistical power and reduce bias associated with attrition.

### Ethical Considerations

Ethical approval for the study was obtained from the Institutional Ethics Committee of Wuhan College (approval #20240306). Prior to each survey administration, students and their parents (or legal guardians) were fully informed about the purpose of the study, data collection procedures, and measures taken to guarantee confidentiality. Parents/guardians and students provided written informed consent. Participation was strictly voluntary, and both students and parents/guardians were assured that refusal or withdrawal would not have any negative consequences on the students’ academic records or school evaluations. To enable longitudinal matching of responses across waves, students were asked to provide their school IDs, which were encrypted, stored securely, and removed from the analytical dataset once the linkage was completed, thereby preserving anonymity. An information sheet describing participants’ rights was distributed prior to survey completion. Participants did not receive any financial compensation for participation. No identifiable images or personal information of participants are included in the manuscript or Multimedia Appendix 1.

### Measures and Covariates

#### Background Variables

Background information collected included age, sex, self-reported academic performance, parental educational level, perceived family financial situations, single-parent family status, and gaming time.

#### IGD Symptoms

IGD symptoms were measured using the nine-item *Diagnostic and Statistical Manual of Mental Disorders, Fifth Edition* (DSM-5) IGD Checklist [2], which assesses the presence of core IGD symptoms over the past 12 months (yes/no response). The nine criteria are preoccupation with gaming, withdrawal, tolerance, loss of control, giving priority to gaming over other activities, persistence despite negative consequences, deception regarding gaming time, escapism, and functional impairment due to gaming. Although the DSM-5 proposes a diagnostic threshold of five or more symptoms for identifying probable IGD, this study treated IGD as a continuous indicator of symptom severity, which is common practice in population-based research examining variability in problematic gaming behaviors [3-7]. The Chinese version of this checklist has been validated among adolescents and has demonstrated satisfactory psychometric properties [69]. Cronbach α was .70 at T1, .75 at T2, and .81 at T3 in this study.

#### Loneliness

Loneliness was assessed using the three-item short form of the University of California, Los Angeles (UCLA) Loneliness Scale [70]. This brief measure captures core aspects of subjective social isolation, including feelings of lacking companionship, being left out, and experiencing isolation. Items were rated on a 4-point Likert scale ranging from 1 (never) to 4 (often), with higher scores reflecting greater loneliness. Previous research has demonstrated satisfactory psychometric properties of this instrument among Chinese adolescents [42]. Cronbach α was .86 at T1, .88 at T2, and .91 at T3 in this study.

#### Physical Activity

PA was assessed using the Physical Activity Rating Scale-3 (PARS-3), a three-item self-report instrument evaluating exercise intensity, duration, and frequency [71]. Each dimension is rated on a 5-point scale, and a composite score is derived according to the following formula: exercise intensity × (exercise duration – 1) × exercise frequency. This yields a possible range from 0 to 100. Higher total scores correspond to greater engagement in PA. Previous research has provided evidence of satisfactory psychometric properties of the scale among Chinese populations [26]. Cronbach α was .76 at T1, .79 at T2, and .80 at T3 in this study.

#### Covariates

In the RI-CLPM, sex, age, self-reported academic performance, perceived family financial situation, and single-parent family status were included as time-invariant covariates at the between-person level, whereas weekly gaming time was included as a time-varying covariate at the within-person level.

### Data Analysis

#### Preliminary Analyses

Attrition analyses were performed to examine potential differences between participants who completed all waves and those lost to follow-up, using chi-square tests for categorical variables and independent-sample *t* tests for continuous variables. Pearson correlations were computed to examine bivariate associations among the main study variables. For descriptive statistics and correlation analyses, listwise deletion was applied to handle missing data.

#### Measurement Invariance

Longitudinal measurement invariance was tested for PA, loneliness, and IGD symptoms. Configural invariance was first evaluated to establish a baseline model with acceptable fit, indicated by comparative fit index (CFI)≥0.90, and both root mean square error of approximation (RMSEA) and standardized root mean square residual (SRMR)≤0.08 [72]. Subsequent tests of metric and scalar invariance were based on changes in fit indices, with ΔCFI≤0.01 and ΔRMSEA≤0.015 indicating invariance across the three waves [72]. In addition, measurement invariance across sex was examined using the same sequential procedure (configural, metric, and scalar invariance) to ensure that the constructs were comparable between males and females.

#### Random Intercept Cross-Lagged Panel Modeling

An RI-CLPM was used to examine the within-person associations among PA, loneliness, and IGD symptoms. All variables were treated as continuous variables. Model construction began with a fully unconstrained specification, in which all autoregressive and cross-lagged pathways were estimated freely. Constraints were then introduced by equating autoregressive and cross-lagged paths across adjacent waves to test whether the longitudinal effects were stable over time. Once the optimal model fit was identified, covariates were included in the model. Sex, age, self-reported academic performance, perceived family financial situation, and single-parent family status were included as time-invariant covariates on the random intercepts (RIs), while weekly gaming time was included as a time-varying covariate at the within-person level. Model fit was evaluated using multiple indices, including *χ*^2^(*df*)≤3, both CFI and Tucker-Lewis index (TLI)≥0.90, and both RMSEA and SRMR≤0.08 [73].

#### Multigroup Analysis

To explore potential sexual differences in these associations, a multigroup RI-CLPM was conducted. A series of models, each constraining a specific path, were compared to an unconstrained model in which all paths were freely estimated. Significant differences between sexes were identified using Wald tests (*P*<.05).

#### Software and Missing Data Handling

Preliminary analyses were conducted in IBM SPSS 26.0, and all SEM analyses were performed in Mplus 8.3 using robust maximum likelihood estimation (MLR). Prior to the main analyses, Little’s Missing Completely at Random (MCAR) test was conducted to examine the missing data mechanism. The results indicated that the missing data were consistent with the assumption of MCAR (*χ*²_18_=12.30, *P*=.83). Therefore, missing data were handled using the full information maximum likelihood (FIML) method, which uses all available information to produce unbiased parameter estimates under MCAR assumptions. The FIML method provides unbiased parameter estimates by using all available information, thereby maximizing statistical power [74]. Statistical significance was defined as two-tailed *P*<.05.

## Results

### Attrition Analyses

**Figure 1:**
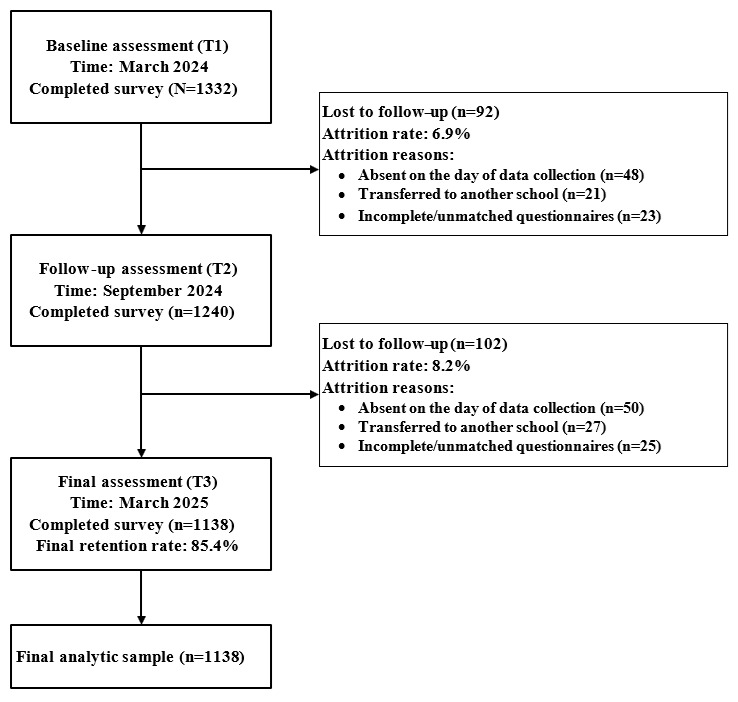
Participant flow diagram across the three waves of the study.

A detailed overview of participant enrollment, retention, and attrition across each wave is provided in Figure 1.

As presented in Table 1, the two groups did not differ significantly with respect to self-reported academic performance, parental education, perceived family financial status, single-parent household status, weekly gaming time, PA, or loneliness. However, significant group differences were observed for sex (*P*=.03), age (*P*=.001), and IGD symptoms (*P*=.02).

**Table 1:** Attrition analyses comparing adolescent gamers who completed all three waves of the longitudinal study and those lost to follow-up (N=1332)^a^.

Characteristics			Follow-up (n=1138)		Lost to follow-up (n=194)		*P* value	
**Sex, n (%)**								.03
	Male	745 (65.6)		142 (73.6)		—^b^		
	Female	390 (34.4)		51 (26.4)		—		
**Self-reported academic performance, n (%)**								
	Bottom 20%	168 (14.8)		18 (9.3)		.19		
	21st-40th percentile	241 (21.2)		41 (21.1)		—		
	41st-60th percentile (average)	288 (25.3)		53 (27.3)		—		
	61st-80th percentile	259 (22.8)		42 (21.6)		—		
	Top 20%	182 (16.0)		40 (20.6)		—		
**Father’s educational level, n (%)**								.05
	Middle school or below	640 (56.2)		105 (54.1)		—		
	High school	284 (25.0)		66 (34.0)		—		
	College or above	214 (18.8)		23 (11.9)		—		
**Mother’s educational level, n (%)**								.84
	Middle school or below	582 (51.1)		98 (50.5)		—		
	High school	351 (30.8)		60 (30.9)		—		
	College or above	205 (18.1)		36 (18.6)		—		
**Perceived family financial situation, n (%)**								.65
	Very poor/poor	149 (13.1)		30 (15.5)		—		
	Average	782 (68.7)		128 (66.0)		—		
	Good/very good	207 (18.2)		36 (18.5)		—		
**Single-parent family status, n (%)**								.58
	No	863 (75.8)		151 (77.8)		—		
	Yes	84 (7.4)		16 (8.2)		—		
	Not reported	191 (16.8)		27 (13.9)		—		
**Gaming time per week** **(hours)** **, n (%)**								.55
	<1	123 (10.8)		20 (10.3)		—		
	1-2	253 (22.2)		44 (22.7)		—		
	2-3	314 (27.6)		55 (28.4)		—		
	3-4	256 (22.5)		42 (21.6)		—		
	>4	192 (16.9)		33 (17.0)		—		
Age (years), mean (SD)			13.61 (0.76)		13.81 (0.71)		.001	
PA^c^ (score 0-100), mean (SD)			75.27 (27.46)		74.35 (27.99)		.67	
Loneliness (score 3-12), mean (SD)			4.61 (2.21)		4.88 (2.07)		.12	
IGD^d^ (score 0-9), mean (SD)			1.57 (1.76)		1.90 (1.86)		.02	

^a^Mean (SD) for continuous variables and n (%) for categorical variables.

^b^Not applicable.

^c^PA: physical activity.

^d^IGD: internet gaming disorder.

### Pearson Correlations

Table 2 demonstrates the bivariate correlations. IGD symptoms were consistently and negatively associated with PA across all three waves, with correlation coefficients ranging from –0.26 to –0.19. Similarly, loneliness exhibited significant negative correlations with PA at each wave, with r values between –0.44 and –0.25. In contrast, IGD symptoms and loneliness were positively correlated over time, with coefficients ranging from 0.17 to 0.29.

**Table 2:** Pearson correlation analyses between PA^a^, loneliness, and IGD^b^ symptoms among adolescents.

Number	Variable	1	2	3	4	5	6	7	8	9
1	PA at T1^c^	1	—^d^	—	—	—	—	—	—	—
2	PA at T2^e^	0.42^f^	1	—	—	—	—	—	—	—
3	PA at T3^g^	0.37^f^	0.43^f^	1	—	—	—	—	—	—
4	Loneliness at T1	–0.39^f^	–0.27^f^	–0.26^f^	1	—	—	—	—	—
5	Loneliness at T2	–0.29^f^	–0.36^f^	–0.27^f^	0.58^f^	1	—	—	—	—
6	Loneliness at T3	–0.25^f^	–0.32^f^	–0.44^f^	0.48^f^	0.59^f^	1	—	—	—
7	IGD symptoms at T1	–0.23^f^	–0.19^f^	–0.21^f^	0.25^f^	0.17^f^	0.19^f^	1	—	—
8	IGD symptoms at T2	–0.23^f^	–0.23^f^	–0.22^f^	0.24^f^	0.27^f^	0.29^f^	0.51^f^	1	—
9	IGD symptoms at T3	–0.25^f^	–0.21^f^	–0.26^f^	0.20^f^	0.25^f^	0.29^f^	0.44^f^	0.55^f^	1

^a^PA: physical activity.

^b^IGD: internet gaming disorder.

^c^T1: wave 1.

^d^Not applicable.

^e^T3: wave 2.

^f^*P*<.001.

^g^T4: wave 3.

### Measurement Invariance Test

Table 3 presents the results of longitudinal measurement invariance testing for PA, loneliness, and IGD symptoms. The configural invariance models showed acceptable fit, with the CFI and TLI exceeding 0.90 and the RMSEA and SRMR below 0.08, indicating that the basic factor structures of these constructs were stable across the three waves. Metric invariance was supported, as changes in fit indices (ΔCFI≤0.01; ΔRMSEA≤0.015) fell within recommended thresholds, suggesting that the factor loadings remained consistent over time. Scalar invariance was also established, with minimal declines in model fit (ΔCFI≤0.01; ΔRMSEA≤0.015), indicating equivalence of item intercepts across measurement occasions. In addition, measurement invariance across sexes was examined using the same sequential procedure (configural, metric, and scalar invariance). The results similarly supported measurement invariance between males and females, indicating that the constructs were comparable across sexes. Detailed results of measurement invariance tests across sexes are presented in Table S1 in Multimedia Appendix 1.

**Table 3:** Longitudinal invariance test of PA^a^, loneliness, and IGD^b^ symptoms.

Variable and model		CFI^c^	TLI^d^	RMSEA^e^	SRMR^f^	ΔCFI	ΔRMSEA	ΔSRMR
**PA**								
	Configural invariance	0.954	0.947	0.038	0.033	—^g^	—	—
	Metric invariance	0.952	0.939	0.042	0.035	0.002	0.004	0.002
	Scalar invariance	0.951	0.937	0.042	0.038	0.001	0.002	0.003
**Loneliness**								
	Configural invariance	0.996	0.989	0.044	0.021	—	—	—
	Metric invariance	0.996	0.992	0.038	0.023	0.000	0.006	0.002
	Scalar invariance	0.995	0.992	0.038	0.023	0.001	0.000	0.000
**IGD symptoms**								
	Configural invariance	0.941	0.929	0.036	0.034	—	—	—
	Metric invariance	0.936	0.927	0.036	0.038	0.005	0	0.004
	Scalar invariance	0.931	0.925	0.039	0.04	0.005	0.003	0.002

^a^PA: physical activity.

^b^IGD: internet gaming disorder.

^c^CFI: comparative fit index.

^d^TLI: Tucker-Lewis index.

^e^RMSEA: root mean square error of approximation.

^f^SRMR: standardized root mean square residual.

^g^Not applicable.

### RI-CLPM Results

**Figure 2:**
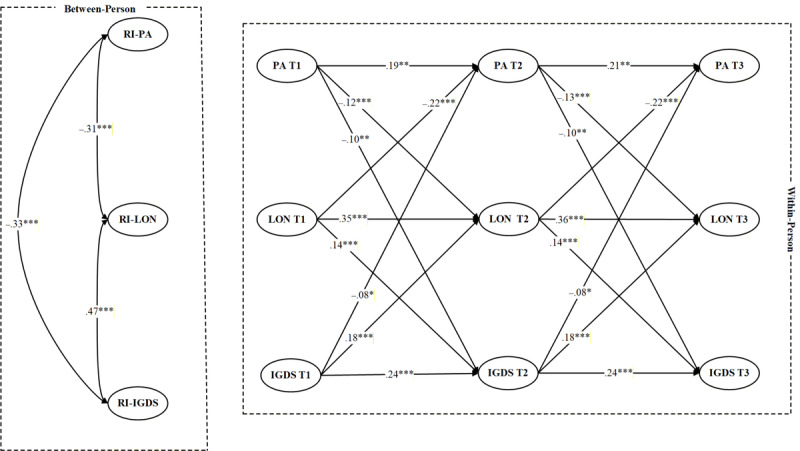
Results of the RI-CLPM (missing data handled via the FIML approach). Standardized estimates were reported. FIML: full information maximum likelihood; IGDS: internet gaming disorder symptoms; LON: loneliness; PA: physical activity; RI-CLPM: random intercept cross-lagged panel model; RI-IGDS: random intercept of internet gaming disorder symptoms; RI-LON: random intercept of loneliness; RI-PA: random intercept of physical activity. *P<.05, **P<.01, ***P<.001.

A fully unconstrained model showed acceptable fit to the data (*χ*²_8_=35.12, CFI=0.985, TLI=0.974, RMSEA=0.043, SRMR=0.030). Subsequently, a model constraining autoregressive and cross-lagged paths to be equal across adjacent waves was estimated. The chi-square difference test indicated that imposing these equality constraints does not significantly worsen model fit (Δ*χ*²_9_=15.35, *P*=.08). Therefore, the more parsimonious constrained model was retained as the final model (*χ*²_17_=50.47, CFI=0.989, TLI=0.977, RMSEA=0.042, SRMR=0.030). The results of model fit comparison are presented in Table S2 in Multimedia Appendix 1. The results of the RI-CLPM examining longitudinal associations among PA, loneliness, and IGD symptoms are summarized in Figure 2.

Autoregressive paths for each variable were significant across waves, indicating substantial temporal stability for PA, loneliness, and IGD symptoms. Cross-lagged effects revealed significant reciprocal associations among PA, loneliness, and IGD symptoms across the three measurement waves. Specifically, higher PA predicted decreased subsequent loneliness (T1→T2: β=–0.12, 95% CI −0.16 to −0.08, *P*<.001; T2→T3: β=–0.13, 95% CI −0.17 to −0.08, *P*<.001), whereas increased loneliness predicted lower PA at the following waves (T1→T2: β=–0.22, 95% CI −0.26 to −0.18, *P*<.001; T2→T3: β=–0.22, 95% CI −0.26 to −0.18, *P*<.001). Similarly, higher PA was associated with decreased subsequent IGD symptoms (T1→T2: β=–0.10, 95% CI −0.16 to −0.04, *P*=.009; T2→T3: β=–0.10, 95% CI −0.16 to −0.04, *P*=.009), while increased IGD symptoms predicted decreases in PA over time (T1→T2: β=–0.08, 95% CI −0.12 to −0.04, *P*=.02; T2→T3: β=–0.08, 95% CI −0.12 to −0.04, *P*=.02). In addition, increased loneliness predicted increases in subsequent IGD symptoms (T1→T2: β=0.14, 95% CI 0.11-0.17, *P*<.001; T2→T3: β=0.14, 95% CI 0.11-0.17, *P*<.001), and increased IGD symptoms, in turn, predicted increased loneliness at the next wave (T1→T2: β=0.18, 95% CI 0.14-0.22, *P*<.001; T2→T3: β=0.18, 95% CI 0.14-0.22, *P*<.001).

Within-person residual correlations revealed that PA was negatively associated with both loneliness and IGD symptoms at all three waves, with standardized coefficients ranging from –0.33 to –0.28 (all *P*<.001). In contrast, loneliness and IGD symptoms were positively correlated at the within-person level across waves, with coefficients ranging from 0.21 to 0.38 (all *P*<.001).

At the between-person level, latent trait correlations further supported these associations. The latent factor of PA was negatively associated with the latent traits of loneliness (r=–0.31, 95% CI –0.45 to –0.17, *P*<.001) and IGD symptoms (r=–0.33, 95% CI –0.49 to –0.17, *P*<.001), indicating that adolescents with higher overall levels of PA across the study period tended to report consistently decreased loneliness and IGD symptoms. Moreover, loneliness and IGD symptoms were strongly and positively associated at the between-person level (r=0.47, 95% CI 0.35-0.59, *P*<.001), suggesting a stable covariation between these two constructs over time.

The effects of time-invariant covariates on the RIs are reported in Table S3 in Multimedia Appendix 1. Sex was significantly associated with the RI of PA (β=−0.08, 95% CI −0.13 to −0.02, *P*=.005) and IGD symptoms (β=−0.08, 95% CI −0.15 to −0.01, *P*=.001), indicating that females (0=male, 1=female) reported slightly lower overall levels of PA and IGD symptoms across the study period. In contrast, age, self-reported academic performance, perceived family financial situation, and single-parent family status were not significantly associated with the RIs (all *P*>.05).

The time-varying effects of gaming time on PA, loneliness, and IGD symptoms are presented in Table S4 in Multimedia Appendix 1. Gaming time was negatively associated with PA at each wave (T1: β=−0.11, 95% CI −0.19 to −0.03, P<.001; T2: β=−0.09, 95% CI −0.15 to −0.03, P=.001; T3: β=−0.06, 95% CI −0.10 to −0.02, P=.04), suggesting that higher gaming engagement is concurrently associated with lower PA. In addition, gaming time was positively associated with loneliness at each wave (T1: β=0.07, 95% CI 0.01-0.13, *P*=.009; T2: β=0.09, 95% CI 0.03-0.15, *P*=.001; T3: β=0.06, 95% CI 0.02-0.10, *P*=.02), suggesting that higher gaming engagement is concurrently associated with increased loneliness. Moreover, gaming time was positively associated with IGD symptoms at each wave (T1: β=0.10, 95% CI 0.04-0.16, *P*<.001; T2: β=0.06, 95% CI 0.01-0.12, *P*=.01; T3: β=0.08, 95% CI 0.02-0.14, *P*=.004), suggesting that higher gaming engagement is concurrently associated with increased IGD symptom severity.

### Sexual Differences

**Figure 3:**
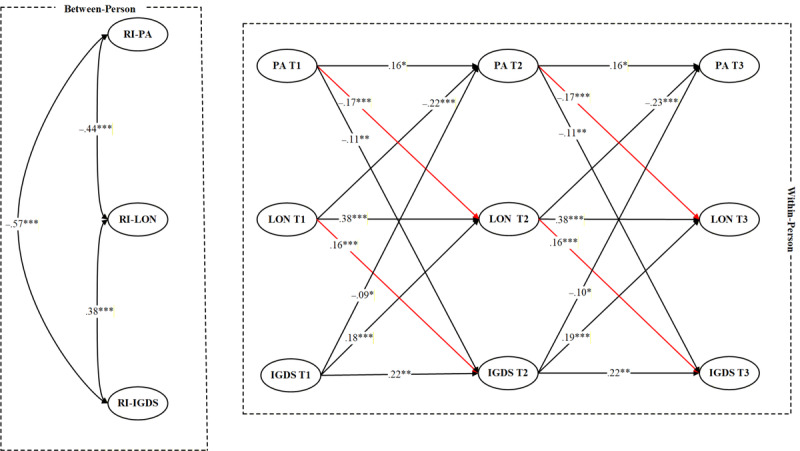
Results of the RI-CLPM for male adolescent gamers (missing data handled via the FIML approach). Standardized estimates were reported. FIML: full information maximum likelihood; IGDS: internet gaming disorder symptoms; LON: loneliness; PA: physical activity; RI-CLPM: random intercept cross-lagged panel model; RI-IGDS: random intercept of internet gaming disorder symptoms; RI-LON: random intercept of loneliness; RI-PA: random intercept of physical activity. *P<.05, **P<.01, ***P<.001.

**Figure 4:**
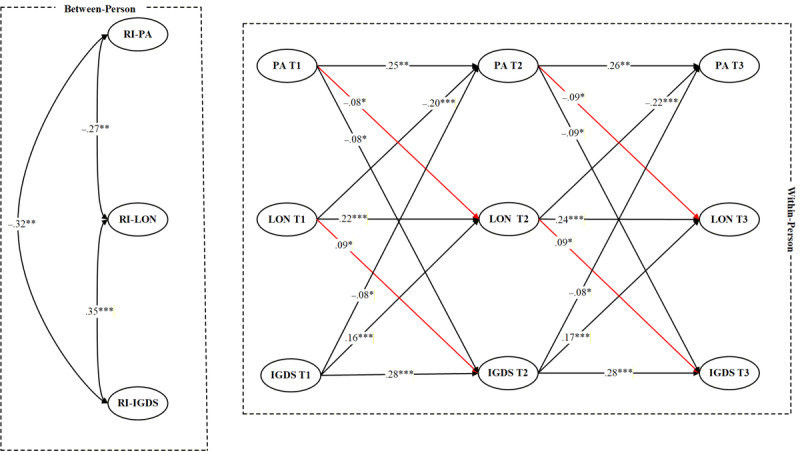
Results of the RI-CLPM for female adolescent gamers (missing data handled via the FIML approach). Standardized estimates were reported. FIML: full information maximum likelihood; IGDS: internet gaming disorder symptoms; LON: loneliness; PA: physical activity; RI-CLPM: random intercept cross-lagged panel model; RI-IGDS: random intercept of internet gaming disorder symptoms; RI-LON: random intercept of loneliness; RI-PA: random intercept of physical activity. *P<.05, **P<.01, ***P<.001.

Multigroup RI-CLPM was conducted to examine whether the longitudinal associations among PA, loneliness, and IGD symptoms varied by sex. In the first step, an unconstrained model allowing all cross-lagged and autoregressive paths to vary freely across males and females was estimated and demonstrated acceptable model fit: *χ*²_8_=21.46, CFI=0.971, TLI=0.954, RMSEA=0.038, SRMR=0.036. In the second step, a constrained model was tested in which all cross-lagged paths were set equal across groups. This model also exhibited good fit: *χ*²_18_=60.24, CFI=0.942, TLI=0.915, RMSEA=0.077, SRMR=0.021. However, the chi-square difference test revealed a significant decline in model fit when the cross-lagged paths were constrained (Δ*χ*²_10_=38.78, *P*<.001), suggesting that the model results significantly differed by sex. Figures 3 and 4 present the results.

Specifically, sexual differences emerged in the path from PA to loneliness. A Wald test of parameter constraints indicated a significant difference between females and males (Wald test estimate=6.27, *P*=.01). For males, PA significantly predicted loneliness (T1→T2: β=–0.08, 95% CI −0.15 to −0.01, *P*=.03; T2→T3: β=–0.09, 95% CI −0.16 to −0.02, *P*=.01). For females, the cross-lagged effects from PA to loneliness were significant but stronger (T1→T2: β=–0.17, 95% CI −0.23 to −0.11, *P*<.001; T2→T3: β=–0.17, 95% CI −0.23 to −0.11, *P*<.001). Sexual differences were also found in the pathway from loneliness predicting IGD symptoms (Wald test estimate=5.87, *P*=.02). Among males, loneliness significantly predicted IGD symptoms (T1→T2: β=0.09, 95% CI 0.03-0.15, *P*=.02; T2→T3: β=0.09, 95% CI 0.03-0.15, *P*=.02). Among females, the paths from loneliness to later IGD symptoms were also significant (T1→T2: β=0.16, 95% CI 0.11-0.21, *P*<.001; T2→T3: β=0.16, 95% CI 0.11-0.21, *P*<.001).

For the remaining cross-lagged paths, no significant sexual differences were observed. Specifically, PA negatively predicted subsequent IGD symptoms for both males (T1→T2: β=−0.08, 95% CI −0.15 to −0.01, *P*=.02; T2→T3: β=−0.09, 95% CI −0.16 to −0.02, *P*=.01) and females (T1→T2: β=−0.11, 95% CI −0.17 to −0.05, *P*=.004; T2→T3: β=−0.11, 95% CI −0.17 to −0.05, *P*=.004). IGD symptoms also predicted decreases in PA across time among males (T1→T2: β=−0.08, 95% CI −0.14 to −0.02, *P*=.03; T2→T3: β=−0.09, 95% CI −0.15 to −0.03, *P*=.02) and females (T1→T2: β=−0.09, 95% CI −0.15 to −0.03, *P*=.02; T2→T3: β=−0.08, 95% CI −0.14 to −0.02, *P*=.03). In addition, IGD symptoms predicted subsequent increases in loneliness among both males (T1→T2: β=0.16, 95% CI 0.11-0.21, *P*<.001; T2→T3: β=0.17, 95% CI 0.12-0.22, *P*<.001) and females (T1→T2: β=0.18, 95% CI 0.13-0.23, *P*<.001; T2→T3: β=0.19, 95% CI 0.14-0.24, *P*<.001). Likewise, loneliness predicted later decreases in PA for males (T1→T2: β=−0.20, 95% CI −0.25 to −0.15, *P*<.001; T2→T3: β=−0.21, 95% CI −0.26 to −0.16, *P*<.001) and females (T1→T2: β=−0.22, 95% CI −0.27 to −0.17, *P*<.001; T2→T3: β=−0.23, 95% CI −0.28 to −0.18, *P*<.001).

### Sensitivity Analyses

To ensure that the main findings were not influenced by missing data or analytic choices, additional analyses were carried out. A complete case analysis (listwise deletion approach) was applied, including only participants who participated in all three assessment waves (n=1138, 85.4%). Although this reduced the sample size, the pattern and size of the cross-lagged paths among PA, loneliness, IGD symptoms were almost identical to those from the FIML model. The similar pattern also appeared in the sex-specific models, indicating that the main conclusions were not sensitive to data exclusion. Figures S1-S3 in Multimedia Appendix 2 present these results. This procedure follows current recommendations for longitudinal research and aligns with previous applications of RI-CLPMs in developmental studies [75,76].

## Discussion

### Principal Findings

This study used a three-wave RI-CLPM to investigate the within-person associations between PA, loneliness, and IGD symptoms among Chinese adolescent gamers. The results revealed bidirectional and longitudinal relationships between PA, loneliness, and IGD symptoms. Multigroup RI-CLPM analyses indicated that the effect of PA on loneliness is stronger among females and that the association between loneliness and subsequent IGD symptoms is also stronger for females. These findings highlight the dynamic nature of these associations, emphasizing the importance of considering temporal and sex-specific patterns in understanding fluctuations in these behaviors over time.

#### Reciprocal Association Between PA and IGD Symptoms

This study revealed clear bidirectional within-person associations between PA and IGD symptoms across time, supporting H1. Consistent with previous studies, this pattern indicates a mutually reinforcing process between PA and IGD symptoms [28,77]. Importantly, the cross-lagged effect size, although statistically significant, was modest (0.08-0.10), aligning with recent methodological benchmarks proposed by Orth et al [78], who noted that cross-lagged effects in longitudinal models are typically small, with β=0.03, 0.07, and 0.12 representing small, medium, and large effects, respectively. From this perspective, the observed effects, although modest, indicate meaningful within-person short-term dynamics across waves without implying long-term accumulation, which is beyond the RI-CLPM framework [79].

The finding that PA prospectively predicts decreased IGD symptoms suggests that engaging in PA may reduce adolescents’ risk for problematic gaming by providing structured, rewarding offline activities that compete with gaming involvement [80]. Participation in team or group-based sports also satisfies needs for relatedness and competence, offering offline sources of reward and belonging that may otherwise be sought in gaming environments [81,82]. In addition, PA may support emotion regulation and self-control, reducing reliance on gaming as a coping strategy [83,84]. The reverse pathway was also observed, with increased IGD symptoms predicting lower subsequent PA, suggesting that problematic gaming may constrain adolescents’ active engagement [85]. As gaming time expands, it may displace opportunities for physical movement, disturb daily routines, and foster social withdrawal from offline peer contexts, collectively diminishing the motivation and energy required for regular exercise [86]. These within-person reciprocal associations suggest a short-term reinforcing cycle between PA and IGD symptoms.

By applying an RI-CLPM, the study clarified that these associations reflect within-person temporal dynamics rather than stable between-person differences. These findings highlight the potential value of promoting regular PA as part of strategies aimed at reducing problematic gaming among adolescents [87]. Importantly, sustaining increases in PA over longer periods can be challenging among adolescents. Therefore, interventions may benefit from combining PA promotion with additional strategies, such as structured reductions in gaming time, parental monitoring, and school- or community-based programs that encourage balanced leisure activities [88,89].

#### Reciprocal Association Between Loneliness and IGD Symptoms

This study revealed consistent bidirectional within-person associations between loneliness and IGD symptoms over time, supporting H2. The finding that loneliness predicts increased IGD symptoms is consistent with compensatory internet use theory [34]. Online gaming may provide immediate gratification, social interaction, and emotional relief, temporarily alleviating feelings of loneliness [90]. However, as adolescents continue to seek solace in gaming, it can lead to more persistent problematic gaming behaviors. This process may be further compounded by the lack of emotional regulation that often accompanies loneliness, which leaves adolescents more vulnerable to maladaptive coping strategies, such as excessive gaming [91]. Equally important is the reverse path: increased IGD symptoms predicting increased loneliness over time. Excessive gaming may reduce offline social interaction, contributing to increased social isolation and loneliness [92,93]. This pattern suggests a reinforcing cycle in which loneliness promotes gaming, while gaming further intensifies loneliness. As problematic gaming often substitutes for meaningful social connections, it further diminishes adolescents’ opportunities to foster real-world relationships [94].

Furthermore, the use of RI-CLPMs clarifies that these associations reflect within-person temporal dynamics rather than stable between-person differences. This approach strengthens the conclusion that interventions targeting loneliness and IGD symptoms need to address their dynamic and reciprocal nature. Consistent with previous research, these findings suggest that interventions should address both emotional distress and gaming behaviors [45]. This highlights the importance of integrating interventions that focus on emotion regulation and social connection, such as promoting offline social activities, while simultaneously addressing problematic gaming behaviors [95]. Strengthening offline social engagement and coping skills may help mitigate the negative impacts of both loneliness and IGD symptoms [96]. As a practical implication, fostering social relationships and coping skills in offline settings, such as through group activities or community support programs, could serve as an effective way to mitigate the negative impacts of both loneliness and IGD symptoms on adolescents [97].

#### Reciprocal Association Between PA and Loneliness

This study revealed clear bidirectional within-person associations between PA and loneliness across time, supporting H3. The reciprocal relationship highlights the importance of understanding how emotional well-being and PA influence each other over time, particularly in adolescence [98]. The finding that PA predicts decreased loneliness is consistent with prior research emphasizing the role of structured, socially engaging activities in alleviating loneliness [58]. PA offers adolescents a sense of competence and achievement, which enhances self-esteem and buffers against emotional distress [83]. This is particularly important during adolescence, a period in which social validation and peer acceptance are key to emotional well-being [99]. Conversely, the finding that loneliness predicts reduced PA suggests that emotional distress plays a significant role in adolescents’ engagement in healthy behaviors. Loneliness can undermine adolescents’ motivation to participate in PA, as the emotional pain of isolation may lead them to withdraw from social settings, including sports or group activities [98]. In line with BPNT, when the need for relatedness is not met, adolescents are less likely to engage in activities that require social interaction, such as PA [100].

From a practical perspective, the findings suggest that promoting regular PA could be an effective strategy for reducing loneliness in adolescents. Interventions that incorporate group-based sports or other socially engaging physical activities could help adolescents build stronger social connections, thereby reducing feelings of isolation [101]. At the same time, addressing loneliness through social support or community-building activities may also encourage adolescents to re-engage in PA, ultimately improving both their emotional well-being and physical health [102]. This highlights the importance of integrating interventions that target both emotional distress (loneliness) and behavioral patterns, such as excessive gaming, while simultaneously promoting PA, to break the self-reinforcing cycle between these factors.

#### Concurrent Associations of Gaming Time With PA, Loneliness, and IGD Symptoms

Gaming time was concurrently linked with adolescents’ PA, loneliness, and IGD symptoms. These patterns suggest that gaming engagement may function as a shared behavioral context in which reduced PA, increased loneliness, and problematic gaming co-occur. This finding aligns with research showing that intensive gaming can restructure adolescents’ daily routines, displacing PA while simultaneously shifting social interaction from offline contexts to online environments [103-105]. Thus, gaming time may represent a proximal behavioral condition that connects lifestyle behaviors and psychosocial experiences, complementing the longer-term within-person dynamics identified by the RI-CLPM analyses.

#### Sexual Differences

Multigroup RI-CLPM analyses revealed significant sexual differences in the paths from PA to loneliness and from loneliness to IGD symptoms, supporting H4. Specifically, the effects of these two pathways were stronger among females. Several potential explanations may help account for these sexual differences. First, sex-related socialization and developmental psychology suggest that adolescent females are more likely to prioritize interpersonal relationships and social connectedness than males [106,107]. As such, feelings of loneliness may elicit more intense emotional distress in females, leading them to seek social compensation through online interactions, including gaming [90]. This is consistent with research indicating that females often experience higher interpersonal sensitivity and engage in more internalizing responses to social stress, which could make them more likely to use gaming or other online avenues to cope with loneliness [5]. Second, the motivations for gaming may differ between sexes. Although males tend to engage in gaming for achievement and competition, females are more likely to seek social affiliation through gaming [108]. Therefore, if females’ gaming is more socially motivated, loneliness may act as a stronger precursor to problematic gaming behaviors in females. Our findings support this pattern, indicating that loneliness functions as a stronger prospective risk factor for IGD symptoms among adolescent females. Similarly, Lacko et al [109] reported sex-specific within-person effects using an RI-CLPM, showing that increases in social gaming were associated with decreased loneliness among boys but increased loneliness among girls. Although their study focused on social gaming rather than IGD symptoms, their findings suggest that gaming-related behaviors may have different psychosocial implications for male and female adolescents.

Practically, these findings underscore the importance of sex-sensitive interventions [110]. For females, interventions should prioritize enhancing social connectedness, providing peer support, and promoting emotion-focused coping strategies alongside PA to address the stronger impact of PA on loneliness [95]. For males, programs may benefit from emphasizing PA as an alternative reward system and offering structured, competitive activities to reduce the appeal of gaming and mitigate its negative impact [95].

### Limitations

This study has several limitations. First, variables, especially PA, were assessed using self-report questionnaires, which may be subject to response biases, such as social desirability or recall bias. Future studies could incorporate multi-informant reports or objective measures (eg, accelerometers) to improve accuracy. Second, although the three-wave design enabled examination of temporal dynamics, the short intervals and limited number of waves restrict the ability to capture longer-term developmental processes. More frequent assessments across longer periods are needed in future studies. Third, although cross-lagged effects were statistically significant and fell within the medium-to-large range [78], their magnitudes should still be interpreted with caution in the light of the large sample size and the reliance on self-reported measures. Fourth, although missing data analyses suggested that the missing-at-random assumption was reasonable and FIML estimation was applied, differential attrition related to sex, age, and IGD symptoms may still introduce potential bias. Fifth, the sample consisted of Chinese adolescent gamers from urban schools, which may limit generalizability to other cultural or contextual settings, particularly rural populations. Future research should consider including more diverse samples and examining potential urban-rural differences. Finally, the study did not collect data on the types of games played (eg, competitive, social, or cooperative), which could have provided additional insights into how different gaming experiences influence the observed outcomes.

### Conclusion

This study used a three-wave longitudinal design to examine the within-person dynamics between PA, loneliness, and IGD symptoms among Chinese adolescent gamers. The results revealed bidirectional relationships between these variables, reflecting a mutually reinforcing cycle. Importantly, sexual differences were observed in the strength of these associations, with the effects of PA on loneliness and loneliness on IGD symptoms being stronger for females. By using an RI-CLPM to distinguish within-person changes from stable between-person differences, this study extends prior research based mainly on cross-sectional designs or traditional CLPMs and provides a clearer understanding of how behavioral and emotional factors jointly shape adolescent problematic gaming. The results suggest that interventions should be sex sensitive, addressing both PA and loneliness simultaneously to effectively mitigate the risk of IGD symptoms, particularly among females.

## Data Availability

The datasets generated and analyzed during the study are available from the corresponding author upon reasonable request.

## Appendix

Multimedia Appendix 1 Measurement invariance across sex; model fit comparison between unconstrained model and constrained model; effects of time-invariant covariates on the random intercepts in the RI-CLPM; and concurrent effects of gaming time on PA, loneliness, and IGD symptoms across the three waves in the RI-CLPM. IGD: internet gaming disorder; PA: physical activity; RI-CLPM: random intercept cross-lagged panel model.

Multimedia Appendix 2 Results of the RI-CLPM for all participants, male adolescent gamers, and female adolescent gamers. RI-CLPM: random intercept cross-lagged panel model.

## Abbreviations

BPNT: basic psychological needs theory
CFI: comparative fit index
CLPM: cross-lagged panel model
DSM-5: Diagnostic and Statistical Manual of Mental Disorders, Fifth Edition
FIML: full information maximum likelihood
IGD: internet gaming disorder
MCAR: Missing Completely at Random
PA: physical activity
RI: random intercept
RI-CLPM: random intercept cross-lagged panel model
RMSEA: root mean square error of approximation
SEM: structural equation modeling
SRMR: standardized root mean square residual
TLI: Tucker-Lewis index
UCLA: University of California, Los Angeles
