# Supplementary material for: Reciprocal Within-Person Dynamics Between Internet Gaming Disorder Symptoms, Physical Activity, and Loneliness Among Chinese Adolescent Gamers: Three-Wave Prospective Cohort Study
Source: JMIR Serious Games. 2026 Apr 27;14:e87847. doi: 10.2196/87847 (PMC13161836; doi:10.2196/87847)
Supplement: Multimedia Appendix 1 [file games_v14i1e87847_app1.docx]

Supplementary Table 1 Measurement invariance across gender

| **Variable** | **Model** | **CFI** | **TLI** | **RMSEA** | **SRMR** | **ΔCFI** | **ΔRMSEA** | **ΔSRMR** |
| --- | --- | --- | --- | --- | --- | --- | --- | --- |
| PA | Configural Invariance | 1.000 | 1.000 | 0.000 | 0.000 | - | - | - |
|  | Metric Invariance | 0.999 | 0.998 | 0.003 | 0.005 | 0.001 | 0.002 | 0.005 |
|  | Scalar Invariance | 0.992 | 0.99 | 0.008 | 0.012 | 0.007 | 0.008 | 0.007 |
|  |  |  |  |  |  |  |  |  |
| Loneliness | Configural Invariance | 1.000 | 0.000 | 0.000 | 0.000 | - | - | - |
|  | Metric Invariance | 0.998 | 0.997 | 0.005 | 0.008 | 0.002 | 0.005 | 0.008 |
|  | Scalar Invariance | 0.994 | 0.989 | 0.015 | 0.012 | 0.004 | 0.008 | 0.004 |
|  |  |  |  |  |  |  |  |  |
| IGD | Configural Invariance | 0.971 | 0.961 | 0.025 | 0.035 | - | - | - |
|  | Metric Invariance | 0.965 | 0.957 | 0.033 | 0.043 | 0.006 | 0.008 | 0.008 |
|  | Scalar Invariance | 0.963 | 0.949 | 0.035 | 0.045 | 0.002 | 0.002 | 0.002 |

PA = physical activity, IGD = internet gaming disorder. CFI = Comparative Fit Index, TLI = Tucker-Lewis index, RMSEA = Root Mean Square Error of Approximation, SRMR = Standardized Root Mean Square Residual.

Supplementary Table 2 Model fit comparison between unconstrained model and constrained model

| Models | χ^2^ | *df* | CFI | TLI | RMSEA | SRMR | Δχ^2^ | Δ*df* | *P* |
| --- | --- | --- | --- | --- | --- | --- | --- | --- | --- |
| Unconstrained model | 35.12 | 8 | 0.985 | 0.974 | 0.043 | 0.03 | 15.35 | 9 | .082 |
| Constrained model | 50.47 | 17 | 0.989 | 0.977 | 0.042 | 0.03 |  |  |  |

Supplementary Table 3 Effects of time-invariant covariates on the random intercepts in the RI-CLPM

| Variable | RI-PA | | |  | RI-Lon | | |  | RI-IGDS | | |
| --- | --- | --- | --- | --- | --- | --- | --- | --- | --- | --- | --- |
|  | *β* (SE) | 95% CI | *P* |  | *β* (SE) | 95% CI | *P* |  | *β* (SE) | 95% CI | *P* |
| Age | -0.02 (0.03) | (-0.08, 0.03) | .416 |  | -0.01 (0.03) | (-0.06, 0.05) | .973 |  | -0.01 (0.03) | (-0.06, 0.04) | .822 |
| Gender | -0.08 (0.03) | (-0.13, -0.02) | .005 |  | 0.05 (0.03) | (-0.01, 0.11) | .074 |  | -0.08 (0.03) | (-0.15, -0.01) | .001 |
| SRAP | -0.03 (0.03) | (-0.08, 0.02) | .251 |  | 0.06 (0.04) | (-0.01, 0.13) | .058 |  | -0.02 (0.03) | (-0.07, 0.03) | .455 |
| PFFS | 0.05 (0.03) | (-0.01, 0.10) | .088 |  | -0.02 (0.03) | (-0.04, 0.01) | .388 |  | -0.02 (0.03) | (-0.07, 0.03) | .481 |
| SPFS | 0.01 (0.03) | (-0.05, 0.06) | .881 |  | 0.01 (0.03) | (-0.04, 0.07) | .629 |  | 0.01 (0.03) | (-0.04, 0.07) | .862 |

SRAP = self-reported academic performance, PFFS = perceived family financial situation, SPFS = single-parent family status. RI denotes the random intercept representing stable between-person differences across waves. RI-PA _=_ random intercept of physical activity, RI-Lon = random intercept of loneliness, RI-IGDS = random intercept of IGD symptoms.

Supplementary Table 4 Concurrent effects of gaming time on PA, loneliness, and IGD symptoms across the three waves in the RI-CLPM

| Variable | PA | | |  | Loneliness | | |  | IGD | | |
| --- | --- | --- | --- | --- | --- | --- | --- | --- | --- | --- | --- |
|  | *β* (SE) | 95% CI | *P* |  | *β* (SE) | 95% CI | *P* |  | *β* (SE) | 95% CI | *P* |
| Gaming time T1 | -0.11 (0.04) | (-0.19, -0.03) | < .001 |  | 0.07 (0.03) | (0.01, 0.13) | .009 |  | 0.10 (0.03) | (0.04, 0.16) | < .001 |
| Gaming time T2 | -0.09 (0.03) | (-0.15, -0.03) | .001 |  | 0.09 (0.03) | (0.03, 0.15) | .001 |  | 0.06 (0.03) | (0.01, 0.12) | .012 |
| Gaming time T3 | -0.06 (0.02) | (-0.10, -0.02) | .043 |  | 0.06 (0.02) | (0.02, 0.10) | .024 |  | 0.08 (0.03) | (0.02, 0.14) | .004 |

PA = physical activity, IGD = internet gaming disorder.
