# Supplementary material for: Reciprocal Within-Person Dynamics Between Internet Gaming Disorder Symptoms, Physical Activity, and Loneliness Among Chinese Adolescent Gamers: Three-Wave Prospective Cohort Study
Source: JMIR Serious Games. 2026 Apr 27;14:e87847. doi: 10.2196/87847 (PMC13161836; doi:10.2196/87847)
Supplement: Multimedia Appendix 2 [file games_v14i1e87847_app2.docx]

**Figure S1.** Results of the RI-CLPM (missing data handled via listwise deletion approach). Standardized estimates were reported. PA = physical activity; LON = loneliness; IGDS = Internet gaming disorder symptoms; RI-PA = random intercept of physical activity; RI-LON = random intercept of loneliness; RI-IGDS = random intercept of Internet gaming disorder symptoms; RI-CLPM = random intercept cross-lagged panel model. **p* < .05, ***p* < .01, ****p* < .001.

**Figure S2.** Results of the RI-CLPM for male adolescent gamers (missing data handled via listwise deletion approach). Standardized estimates were reported. PA = physical activity; LON = loneliness; IGDS = Internet gaming disorder symptoms; RI-PA = random intercept of physical activity; RI-LON = random intercept of loneliness; RI-IGDS = random intercept of Internet gaming disorder symptoms; RI-CLPM = random intercept cross-lagged panel model. **p* < .05, ***p* < .01, ****p* < .001.

**Figure S3.** Results of the RI-CLPM for female adolescent gamers (missing data handled via listwise deletion approach). Standardized estimates were reported. PA = physical activity; LON = loneliness; IGDS = Internet gaming disorder symptoms; RI-PA = random intercept of physical activity; RI-LON = random intercept of loneliness; RI-IGDS = random intercept of Internet gaming disorder symptoms; RI-CLPM = random intercept cross-lagged panel model. **p* < .05, ***p* < .01, ****p* < .001.
